# 

**Published:** 1893-10

**Authors:** 


THE MAGAZINE OF THE FUTURE.—The July *Cosmopolitan* marked the most radical step ever taken in periodical literature. With that issue, the magazine, unchanged in form, in fact one of the best numbers of the *Cosmopolitan* ever issued, was put on sale at twelve and one-half cents per copy—$1.50 a year. The cutting in half of a price already deemed low for an illustrated magazine, is the result of an intention long since formed, to give to the public an illustrated monthly of the very highest class at such a price as must bring it within the reach of all persons of intellectual tastes, however limited their incomes. There are more than 10,000,000 readers in the United States, and less than 800,000 magazines are printed to supply their demands. More than four years have been spent in reaching the organization necessary for the production of the *Cosmopolitan* at this price, a figure hitherto undreamed of by the reading world. Each department of the work has been slowly perfected, until, with the January number of this year, 150,000 copies of the magazine were prepared upon presses and machinery of the most improved form, built with a view to producing the finest results at the very minimum of expense—the only establishment in the world, it is believed, devoted exclusively to the printing of an illustrated monthly magazine. To establish a magazine upon such a basis at the outset was impossible. Only the rapid growth of the *Cosmopolitan's* editions, almost unprecedented in magazine records, has produced the conditions which make this departure from established prices possible. The *Cosmopolitan* promises to make the year 1893 the most brilliant in its history. No other year has seen such an array of distinguished names as will appear on its title page during 1893. De Maupassant, Mark Twain, George Ebers, Valdez, Spielhagen, Francois

Coppée, Flammarion, and Paul Heyse, are some of the authors whose work will appear for the first time this year in the pages of the *Cosmopolitan*. Among the artists whose work will decorate its pages for the first time during 1893 are Laurens, Toussaint, Vierge, Rochegrosse, and Schwabe. William Dean Howells will be a regular contributor during 1893–94.

---

THE Secretary of the State Board of Health, Dr. Lewis Balch, has prepared a manual for the use of members of local boards of health, health officers, and all others interested in health matters. The book is exactly what it purports to be a—practical working manual. It defines the powers of the State and local boards, it contains directions to the local health officer, it gives examples of problems which may arise and their solution, it offers suggestions for the prevention of disease, and it includes directions to be followed in times of danger from epidemics of contagious diseases which formulate the best method of stamping these out which experience has devised. It solves many legal questions in the most plain and practical way. The value of the vital statistics gathered by the State Board is explained, and the duty of those who are required by the law to fill out the certificates is fully defined. Blank certificates, having the questions properly answered, are given as models to be followed. Bound with the manual is a copy of the Public Health Law, to which it is designed to serve as a commentary. The volume will be found to be of the greatest value to all who are interested in the public health, and it will enable boards of health and health officers to be certain of their positions in their dealings either with their municipal governments or with the people. Price, $1.50; delivered upon receipt of price. Banks & Brothers, Albany, N. Y.

---

A NEW ILLUSTRATED DICTIONARY OF MEDICINE, BIOLOGY, AND COLLATERAL SCIENCES.—Dr. George M. Gould, already well known as the editor of two small medical dictionaries, has now about ready an unabridged, exhaustive work of the same class, upon which he and a corps of able assistants have been uninterruptedly engaged for several years.

The feature that will attract immediate attention is the large number of fine illustrations that have been included, many of which — as, for instance, the series of over fifty of the bacteria—have been drawn and engraved especially for the work. Every scientificminded physician will also be glad to have defined several thousand commonly used terms in biology, chemistry, etc.

The chief point, however, upon which the editor relies for the success of his book, is the unique epitomization of old and new knowledge. It contains a far larger number of words than any other one-volume medical lexicon. It is a new book, not a revision of the older volume. The pronunciation, etymology, definition, illustration, and logical groupings of each word are given. There has never been such a gathering of new words from the living literature of the day. It is especially rich in tabular matter, a method of presentation that focuses, as it were, a whole subject so as to be understood at a glance.

The latest method of spelling certain terms, as adopted by certain scientific bodies and authorities, have all been included, as well as those words classed as obsolete by some editors, but still used largely in the literature of today, and the omission of which in any work aiming to be complete would make it unreliable as an exhaustive work of reference.

The publishers, Messrs. P. Blakiston Son & Co., announce that, notwithstanding the large outlay necessary to its production on such an elaborate plan, the price will be no higher than that of the usual medical text-book.

---

A New Medical Dictionary.—A completely new medical dictionary is announced for early publication by Lea Brothers & Co. The author, Dr. Alexander Duane, of New York, is already widely known as the medical expert for Webster's International Dictionary. His new work has been drafted to supply medical students with all desired information concerning the words they will meet in their course of reading, and as the vocabulary has been selected most liberally, the work will be of value to practitioners also. The pronunciation of each word is given by a simple and obvious phonetic spelling; then follows the derivation, an unexcelled aid to memory, and finally a full definition. Descriptive matter has been appended to such words as cannot be adequately explained by simple definition. Thus, diseases are described, and their symptoms and treatment are given; drugs are followed by their properties, effects, doses, etc. Extensive tables of bacteria, doses, etc., are placed in the alphabet most conveniently for reference. A work of real value is promised, and we shall take an early opportunity of reviewing it in these columns.

Gray's Anatomy, New (Thirteenth) Edition.—Another edition, the thirteenth, of this standard work is announced for early publication by Messrs. Lea Brothers & Co. It is hardly too much to say that this work has been the most popular of all medical text-books whatever since its first appearance, in 1851. Its text has been revised successively by the foremost anatomists of a generation, and the present edition embodies whatever changes were necessary to make it represent its advancing science. The illustrations have always been noted for their clearness. Their large size has rendered it possible to print the names of the parts directly upon them, thereby indicating not only their names, but also their extent—a most important matter. A liberal use of colors has been made to secure additional prominence for certain parts. Notwithstanding these improvements, the constantly increasing demand has justified a reduction in the price of the colored edition. An early review will appear in these columns.

---

A Text-Book of Normal Histology, by Dr. George A. Piersol, Professor of Anatomy in the University of Pennsylvania, is announced by J. B. Lippincott Company, among other new medical publications, for early issue. The author is eminently qualified by learning, research, and experience as a teacher, to prepare such an important work as this, and in the arrangement of the text shows a wise sympathy with the wants of the students. While his descriptions of the various tissues and organs are sufficiently full, he has carefully avoided such detail as would bewilder the learner. The numerous illustrations are excellent, and with few exceptions cover the entire field of normal histology.

---

Dunglison's New Pronouncing Medical Dictionary.—A new edition of Dunglison's Medical Dictionary is announced as in press for early publication. It has been thoroughly revised and greatly enlarged, and will contain about *forty-four thousand* new medical words and phrases. Pronunciation has been introduced into the new edition by means of a simple phonetic spelling. This work has always been noted for the fulness of its definitions, ample explanations being its distinguishing characteristic. In the new edition much encyclopedic information, difficult of access elsewhere, will be found conveniently at hand. Especial attention has been devoted to matters of practical value. A review will appear in an early issue.

Dr. Thomas More Madden, well known as one of the foremost gynecologists of Europe, has prepared a hand-book of Diseases Peculiar to Women, which will be published at once by J. B. Lippincott Company. The work is more than usually important, from the fact that it contains the result of the author's wide experience and is embellished with numerous excellent sketches of gynecological diseases and appliances, together with engravings and drawings or photographs of cases under clinical observation made by some of the most eminent physicians in England and America. The work is entitled Clinical Gynecology.

---

Elinor Fenton, by David S. Foster, is a charming romance, the scene of which is laid in the heart of the Adirondacks. The plot is full of incident, and the characters, manners, and scenery are happily depicted. Love receives a due share of attention, its blisses, its woes, its apparent defects, and ultimate victory being described with much humor in a very life-like way. Mr. Foster has produced a pleasing story that is sure to increase a reputation already recognized. It is published by J. B. Lippincott Company.

---

About October 15th, a Medical Directory of the State of Connecticut will be issued by the Danbury Medical Printing Company, of Danbury, Conn. It will contain a list of all the medical practitioners of the State, the various medical societies, all the dentists and dental societies, druggist and pharmaceutical societies, nurses and training schools for nurses, hospitals, etc. Price, $1.00, delivered free by post.

---

Frank R. Stockton has written the history of "How I Wrote 'The Lady, or the Tiger,'" for the next issue of *The Ladies' Home Journal*, and tells what came of the writing of the famous story, and the condition of his own mind, at the present time, of the correct solution of the problem, whether the lady or the tiger came out of the opened door.

---

Notice to Contributors.—We are glad to receive contributions from every one who knows anything of interest to the profession. Articles designed for publication in the Journal should be handed in before the first day of the month. The Editors are not responsible for the views or opinions of contributors. All communications should be addressed to the Managing Editor, 284 Franklin St., Buffalo, N. Y.

